# Supplementary material for: In Silico Analysis of MiRNA Regulatory Networks to Identify Potential Biomarkers for the Clinical Course of Viral Infections
Source: Int J Mol Sci. 2025 Oct 16;26(20):10100. doi: 10.3390/ijms262010100 (PMC12563699; doi:10.3390/ijms262010100)
Supplement: Supplementary file 1 [file ijms-26-10100-s001.zip › ijms-3890870-supplementary.pdf]

International Journal of *Molecular Sciences*

MDPI

Supplementary

Figure S1. The regulatory network of miRNA-gene interactions involved in viral infections. The following GO genes are represented: 2381, 2889, and 2455

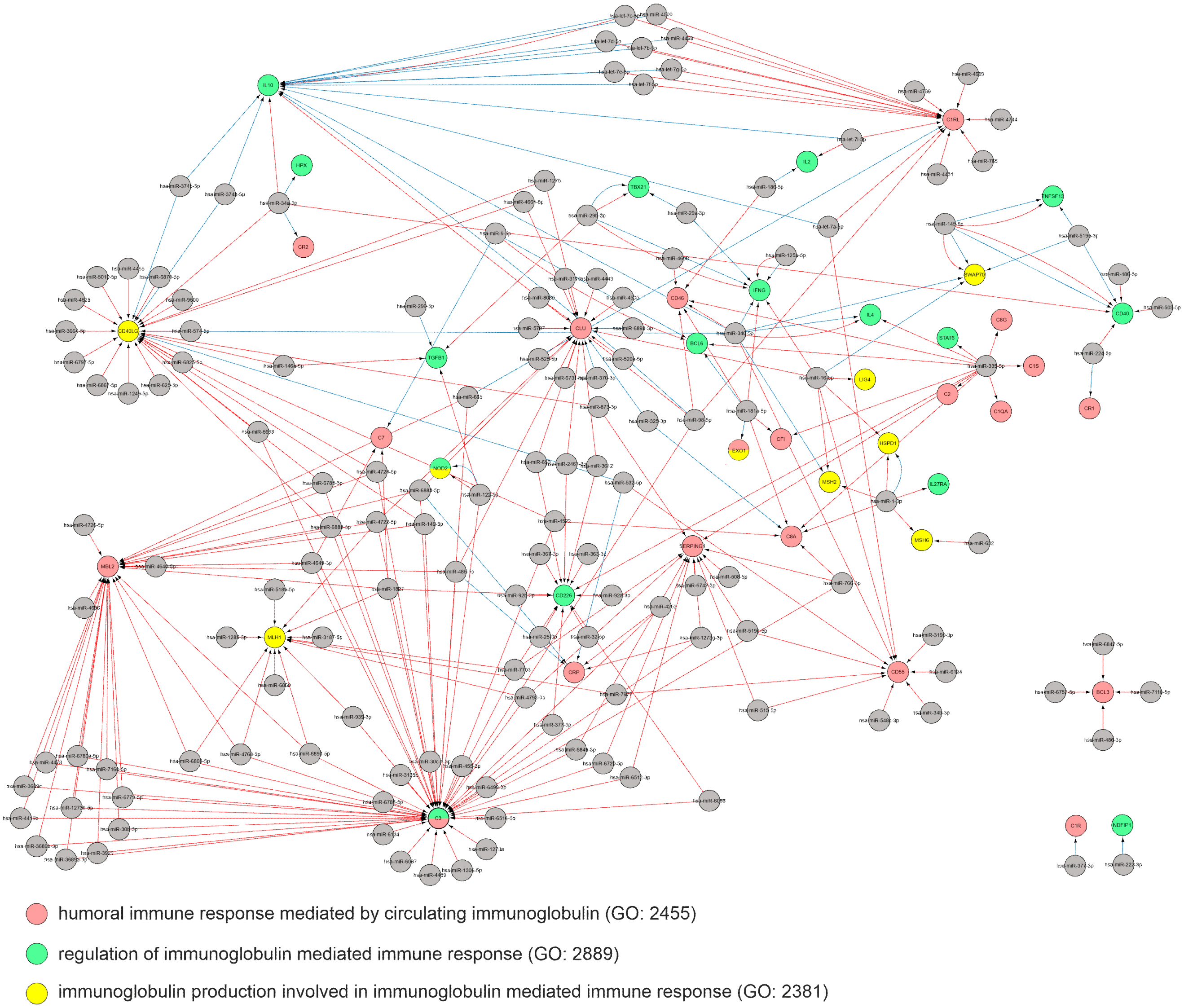

Table. S1. Thermodynamic and structural characteristics of predicted mRNA-miRNA interactions.

	miRNA	Site_Position	LogitProb†	Score	Site_Access* ††	ΔGhybrid †††	ΔGnucl*	ΔGtotal*	Site_Location*
BCL3	hsa-miR-486-3p	65-96	0.355	0.045	0.419	-26.800	-1.787	-1.700	0.152
EOMES	hsa-miR-25-3p	105-131	0.554	0.109	0.363	-16.400	-0.008	-0.219	0.093
EOMES	hsa-miR-29a-3p	770-803	0.541	0.050	0.347	-18.200	-0.396	-2.458	0.678
EOMES	hsa-miR-29b-3p	780-803	0.603	0.034	0.350	-19.000	-0.508	-1.783	0.687
EOMES	hsa-miR-363-3p	36-45	0.853	0.075	0.477	-20.000	-0.791	-14.962	0.032

EOMES	hsa-miR-367-3p	104-132	0.581	0.055	0.379	-20.700	-3.220	-2.783	0.092
ICAM1	hsa-miR-146a-5p	706-717	0.599	0.078	0.454	-16.400	-1.503	-5.235	0.530
ICAM1	hsa-miR-92a-3p	324-336	0.634	0.164	0.357	-19.800	-0.088	-4.734	0.227
ICAM1	hsa-miR-92b-3p	228-238	0.689	0.145	0.283	-17.400	-0.047	-6.924	0.171
ICAM1	hsa-miR-98-5p	1265-1294	0.658	0.084	0.394	-17.900	-0.295	-4.051	0.950
IL10RB	hsa-miR-29b-3p	820-833	0.639	0.039	0.387	-18.400	-1.972	-6.646	0.930
IL1A	hsa-miR-181a-5p	103-132	0.619	0.085	0.386	-17.700	-1.262	-6.027	0.088
IL6	hsa-let-7a-5p	305-322	0.702	0.032	0.286	-18.200	-5.190	-7.557	0.698
IL6	hsa-let-7c-5p	286-322	0.674	0.043	0.307	-21.800	-3.383	-2.662	0.654
IL6	hsa-miR-146a-5p	1736-1750	0.688	0.156	0.506	-15.300	-4.556	-5.512	0.706
IL6R	hsa-let-7a-5p	1989-1998	0.583	0.215	0.490	-17.000	-2.528	-7.242	0.809
IL6R	hsa-let-7c-5p	1973-1998	0.593	0.269	0.656	-19.300	-5.948	-9.875	0.802
IRAK1	hsa-miR-146a-5p	44-63	0.829	0.103	0.265	-26.300	-0.728	-10.453	0.032
PLCG2	hsa-let-7a-5p	349-368	0.659	0.327	0.569	-17.800	-5.682	-9.043	0.074
PLCG2	hsa-let-7c-5p	349-368	0.690	0.409	0.569	-20.800	-5.793	-12.043	0.074
RAB27A	hsa-miR-29a-3p	809-835	0.639	0.072	0.545	-21.900	-0.270	-6.744	0.317
RAB27A	hsa-miR-29b-3p	814-835	0.666	0.070	0.574	-22.000	-3.527	-10.316	0.318
STAT1	hsa-miR-146a-5p	787-794	0.591	0.093	0.174	-16.600	-0.102	-5.078	0.467
STAT3	hsa-miR-181a-5p	253-283	0.575	0.182	0.562	-15.500	-3.478	-3.789	0.100
TRAF6	hsa-miR-146a-5p	1261-1279	0.888	0.140	0.383	-29.500	-2.265	-14.353	0.657

†The "Logistic Probability" parameter demonstrates the confidence in the prediction of the true binding site.

††The "Site Access Score" parameter takes into account the structural accessibility of the predicted binding site on the target mRNA. A high-priority binding site should not only have a high logistic probability but also be structurally accessible. A site with a high probability but low accessibility may be less functional.

†††The calculated changes in hybridization energy (ΔGtotal, ΔGnucl, ΔGhybrid) characterize the interaction (a more negative ΔG value indicates a more stable and preferable interaction).

Features used in the STarMir prediction model are marked with an asterisk (*).
